# Continuation of beta-blockers during prolonged dobutamine infusion in heart transplant–prioritised patients: A competing-risk analysis

**DOI:** 10.1371/journal.pone.0354128

**Published:** 2026-07-21

**Authors:** Joao Manoel Rossi Neto, Victor Bemfica de Mello Mattos, Airton Salviano Sousa Junior, Marco Aurelio Finger, Raphael Machado Rossi, Plinio Jose Whitaker Wolf, Carlo Bonasso Filho, Flavia Potsch Camara Mattos Girard, Carolina Casadei dos Santos

**Affiliations:** Heart Transplant Unit, Instituto Dante Pazzanese de Cardiologia, São Paulo, Brazil; Cleveland Clinic, UNITED STATES OF AMERICA

## Abstract

**Background:**

Patients awaiting heart transplantation (HT) frequently require prolonged continuous dobutamine infusion. Whether oral beta-blocker (BB) therapy should be maintained during this period remains controversial and poorly studied, particularly in the context of prolonged concomitant use. The impact of BB continuation on waiting-list outcomes in this specific scenario is unknown.

**Methods:**

We conducted a retrospective single-centre cohort study including adult patients listed for HT who received ≥ 30 consecutive days of concomitant oral BB therapy and continuous intravenous dobutamine between January 2020 and December 2023. The index date was defined as the first day after completion of this 30-day period, identifying patients who had already demonstrated sustained haemodynamic tolerance to the combination. Patients were classified according to BB continuation or suspension after the index date. Competing-risk analyses were performed using inverse probability of treatment weighting (IPTW), Aalen–Johansen cumulative incidence functions, and Fine–Gray regression. Robustness was assessed through nonparametric bootstrapping (1,500 replications).

**Results:**

Among 53 eligible patients (mean age 50.8 ± 11.9 years; 64% male), 29 underwent HT and 16 died before transplantation. After IPTW adjustment, BB continuation was associated with a higher cumulative incidence of HT at ≥ 270 days (61.1% vs 14.3%; p < 0.001) and a lower cumulative incidence of pre-transplant death (13.3% vs 68.7%; p < 0.001). Fine–Gray regression confirmed these associations: HT sHR 8.79 (95% bootstrap CI 2.66–54.31) and pre-transplant mortality sHR 0.077 (95% bootstrap CI 0.014–0.192). Results were consistent across four pre-specified sensitivity analyses.

**Conclusions:**

This study evaluates outcomes in patients sustaining oral beta-blocker therapy during prolonged dobutamine infusion while awaiting heart transplantation, a clinical scenario with limited available data. BB continuation was associated with a higher probability of transplantation, with consistent direction across pre-specified sensitivity analyses. Given the small sample size and the potential for residual confounding by indication, these findings should be interpreted with caution and are best viewed as hypothesis-generating. They may help inform future research, particularly prospective studies in this setting.

## Introduction

Advanced heart failure (HF) remains a major global health burden consistently associated with high morbidity, mortality, and healthcare utilisation. Heart transplantation (HT) is the definitive therapy for eligible patients; however, donor scarcity and prolonged waiting-list times often necessitate extended periods of in-hospital stabilisation [1–3]. Continuous intravenous dobutamine is frequently required to maintain adequate haemodynamics during this period, despite well-recognised limitations including arrhythmic risk, tachyphylaxis, and increased mortality [4–6]. Identifying strategies that optimise stability and improve waiting-list outcomes in patients dependent on long-term inotropes is therefore of direct clinical relevance, particularly in settings without access to durable mechanical circulatory support.

Beta-blockers (BBs), a cornerstone of chronic HF therapy, provide neurohormonal blockade, reduce arrhythmic burden, and improve long-term outcomes. Their continuation during continuous dobutamine infusion remains controversial, and clinical practice varies widely among transplant centres in the absence of specific guideline recommendations [2,3,7–10]. Concerns regarding pharmacodynamic antagonism and haemodynamic compromise have led many centres to routinely discontinue BB upon inotrope initiation or during acute decompensation.

However, a specific and underexplored clinical scenario exists: patients who have already been receiving oral BB and continuous dobutamine concomitantly for ≥ 30 consecutive days while awaiting transplantation. This population — increasingly encountered in centres where pharmacological bridging is the primary or only available strategy — has not been specifically studied. These patients have, by definition, demonstrated sustained haemodynamic tolerance to the combination, raising the question of whether BB continuation in this adapted state confers prognostic benefit.

Furthermore, prior observational studies examining inotrope–BB interactions have relied on traditional Kaplan–Meier survival analyses, which censor death and consequently overestimate the probability of transplantation [11–13]. Competing-risk methods are required when death precludes the event of interest, and their application in transplant populations has revealed important distinctions in waiting-list trajectories [1,14,15].

We therefore hypothesised that, in patients who had already demonstrated tolerance to prolonged concomitant BB and dobutamine therapy, continuation of BB after the index date would be associated with a higher probability of heart transplantation and reduced pre-transplant mortality, compared with BB suspension.

## Methods

### Study design and setting

We conducted a retrospective, single-centre cohort study at the Instituto Dante Pazzanese de Cardiologia (São Paulo, Brazil). The study included adult patients with advanced HF actively listed for HT who received prolonged, continuous intravenous dobutamine infusion between January 2020 and December 2023. No formal sample size calculation was performed because this was a retrospective study including all eligible patients during the study period.

### Patient selection

Eligible participants were adults (≥ 18 years) listed for HT who received ≥ 30 consecutive days of concomitant oral BB therapy and continuous intravenous dobutamine during hospitalisation. The index date (t = 0) was defined as the first day following completion of this 30-day concomitant period.

The 30-day threshold was selected to define a population of patients who had demonstrated sustained haemodynamic tolerance to combined BB and dobutamine therapy — a duration sufficient to exclude transient pharmacodynamic interactions and identify a clinically stable phenotype of concomitant use. This represents a poorly studied and clinically distinct scenario from acute BB initiation during inotrope therapy, which has been the focus of prior literature.

Exclusion criteria included: (i) prior heart transplantation; (ii) durable mechanical circulatory support before or during the concomitant therapy period; (iii) inactive listing status; and (iv) death or HT prior to completion of the 30-day concomitant period.

### Exposure definition

**BB maintained:** documented continuation of oral BB therapy for ≥ 72 hours after the index date, with no interruption recorded in pharmacy or nursing records.

**BB suspended:** permanent discontinuation of oral BB therapy documented ≥ 72 hours after the index date, occurring in the context of acute haemodynamic deterioration (cardiogenic or septic shock), with no subsequent reinitiation at any point during follow-up.

Follow-up extended until HT, death, or censoring on December 31, 2023.

### Data collection and definitions

Baseline variables collected at the index date included demographics, left ventricular ejection fraction (LVEF), HF aetiology (ischaemic, dilated, Chagas, and other), vital signs, laboratory data (sodium, creatinine, albumin, NT-proBNP, AST [aspartate aminotransferase]), comorbidities (hypertension, diabetes mellitus, dyslipidaemia, chronic kidney disease [CKD]). Medication data included BB class and doses, initial dobutamine dose, renin–angiotensin–aldosterone system inhibitor use (ARNI [angiotensin receptor–neprilysin inhibitor], ACEI [angiotensin-converting enzyme inhibitor], ARB [angiotensin receptor blocker]), mineralocorticoid receptor antagonists (MRA [mineralocorticoid receptor antagonist]), diuretics, and amiodarone.

Variables reflecting the post-index clinical course — including final and mean dobutamine dose, escalation to other vasoactive agents, reason for BB suspension, dialysis initiation, intra-aortic balloon pump (IABP) insertion, and extracorporeal membrane oxygenation (ECMO) initiation — were recorded separately as indicators of haemodynamic deterioration during follow-up. Cardiogenic shock was defined as hypotension (< 90 mmHg systolic or requiring vasoactive support) with evidence of hypoperfusion. Septic shock was defined as sepsis requiring vasopressors to maintain mean arterial pressure ≥ 65 mmHg. No missing data were present for any analysed variable.

### Outcomes

The primary outcome was the cumulative incidence of heart transplantation, treating pre-transplant death as a competing event. The secondary outcome was the cumulative incidence of pre-transplant death, treating HT as the competing event.

### Statistical analysis

Continuous variables were summarised as mean ± SD or median [IQR] and compared using Welch’s t-test or Mann–Whitney U test. Categorical variables were compared using Pearson’s chi-square or Fisher’s exact test.

**Fig 1:**
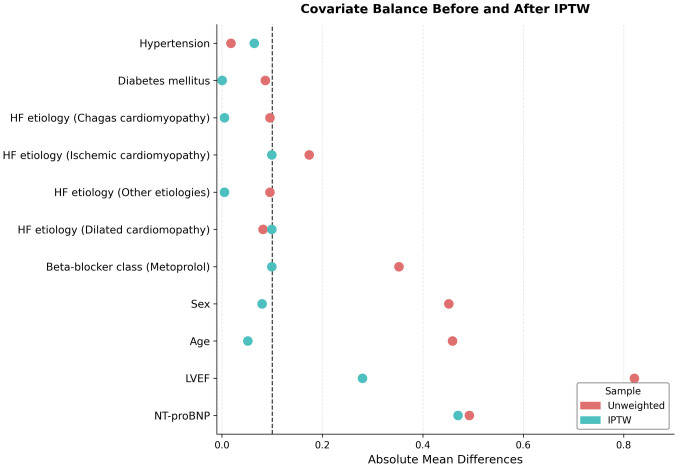
Covariate balance before and after inverse probability of treatment weighting (IPTW). Absolute standardised mean differences (SMDs) are shown for pre-specified baseline covariates before (unweighted, red circles) and after truncated unstabilised ATE IPTW (teal circles). The dashed vertical line indicates the pre-specified threshold for adequate balance (|SMD| = 0.10). IPTW improved covariate balance across most baseline covariates; residual imbalance in LVEF and NT-proBNP persisted after weighting, in the direction of greater disease severity in the BB-suspended group. Abbreviations: IPTW, inverse probability of treatment weighting; SMD, standardised mean difference; ATE, average treatment effect; LVEF, left ventricular ejection fraction; NT-proBNP, N-terminal pro-brain natriuretic peptide; BB, beta-blocker.

To minimise confounding, IPTW was applied based on a parsimonious logistic regression propensity score (PS). The PS included six a priori selected baseline covariates measured at the index date: age, sex, HF aetiology, hypertension, diabetes mellitus, and BB class. Variables reflecting post-index haemodynamic deterioration — dialysis, IABP, dobutamine dose escalation, and shock episodes — were excluded because chart review confirmed these events occurred after the index date; their inclusion would constitute mediator adjustment bias [16]. Furthermore, with only 18 patients in the smaller treatment group and eight estimated model parameters, the events-per-parameter ratio was low (≈ 2.3), well below the conventional minimum of 10; this justified a parsimonious specification, as adding further covariates would have driven the ratio still lower and produced model instability. Accordingly, LVEF and NT-proBNP were considered baseline markers of disease severity and were therefore evaluated separately in covariate-balance diagnostics (Fig 1) and sensitivity analyses (LVEF in a pre-specified multivariable Fine–Gray model) rather than entered into the primary propensity score. Given the limited sample size and low events-per-parameter ratio, inclusion of additional severity markers in the primary propensity model was considered likely to increase the risk of model instability and overfitting [17].

Unstabilised average treatment effect (ATE) weights were calculated as the inverse probability of the observed treatment assignment and truncated at the 1st and 99th percentiles to reduce the influence of extreme observations. Stabilised IPTW weights were examined as a sensitivity analysis and yielded materially similar results (range 0.451–2.164). Covariate balance was evaluated using standardised mean differences (SMDs), with |SMD| < 0.10 indicating adequate balance, and visualised using Love plots. Propensity score distributions by group are shown in Supplementary S1 Fig.

The primary outcome was defined a priori as cumulative incidence of HT at ≥ 270 days; 90-day and 180-day time points were pre-specified exploratory secondary horizons. Cumulative incidence functions (CIFs) were estimated using the Aalen–Johansen estimator. Subdistribution hazard ratios (sHRs) were estimated using Fine–Gray regression, [12]. incorporated through a Fine–Gray weighted Cox formulation with final weights as the product of Fine–Gray censoring weights and unstabilised IPTW. Robust variance was obtained by clustering on individual patient identifiers.

Four pre-specified sensitivity analyses were performed: (i) unweighted Fine–Gray regression; (ii) multivariable Fine–Gray including age, LVEF, and HF aetiology without IPTW; (iii) LASSO-penalised PS with cross-validated regularisation; and (iv) doubly robust Fine–Gray combining IPTW with direct covariate adjustment. Confidence intervals for IPTW-weighted CIF differences were derived from percentile-based nonparametric bootstrap resampling (1,500 replications). To quantify robustness to unmeasured confounding, E-values were calculated using the conservative correction for common outcomes [18]. Full results are reported in Supplementary S2 Table.

Two-sided p-values < 0.05 were considered statistically significant. All analyses were performed using R version 4.4.1 with the cmprsk and survival packages, and Python 3.11.9 (pandas 2.2.2, matplotlib 3.9.0, numpy 1.26.4) for data preprocessing and visualisation.

### Ethics approval

This retrospective study was approved by the Institutional Ethics Committee of the Instituto Dante Pazzanese de Cardiologia (CAAE 77180624.6.0000.5462). In accordance with institutional and national regulations, attempts were made to obtain written informed consent from participants or their legal representatives whenever feasible. When consent could not be obtained, attempts and reasons were documented in patients’ medical records as instructed by the Ethics Committee. No individual patient-identifiable data are disclosed. All procedures complied with the Declaration of Helsinki.

## Results

### Study population

Of the 83 patients screened between January 2020 and December 2023, 30 were excluded: 11 who did not receive dobutamine, 9 aged < 18 years, 1 without beta-blocker use, 1 who died before prioritisation, and 8 who received < 30 days of concomitant therapy. The final cohort comprised 53 patients who completed ≥ 30 consecutive days of combined BB and dobutamine therapy. Among these, 29 underwent HT, 16 died before HT, and 8 were censored at study end.

### Baseline characteristics

The mean age was 50.8 ± 11.9 years and 64% were male. Mean LVEF was 23.0 ± 6.4%. Median hospitalisation duration was 110 days (IQR 74–226). The duration of beta-blocker use after the index date had a median of 101 days (IQR 62–211; maximum 442 days), reflecting the prolonged nature of the clinical scenario studied.

Table 1 presents baseline characteristics at the index date (Panel A) and variables reflecting the post-index clinical course (Panel B). At baseline, groups were broadly similar in LVEF, HF aetiology, comorbidities, BB class, and initial dobutamine dose, with the exception of age and NT-proBNP. During follow-up, BB suspension was strongly associated with escalation of dobutamine dose, requirement for additional vasoactive agents, cardiogenic or septic shock, dialysis initiation, and IABP insertion — all events that developed after the index date, reflecting progressive haemodynamic deterioration rather than pre-existing baseline differences.

**Table 1:** Baseline and post-index clinical characteristics by beta-blocker exposure group.

Variable	BB Suspended (n = 18)	BB Maintained (n = 35)	p-value
**Panel A — Baseline characteristics at index date**			
Age (years)	46.9 [40.0–55.0]	55.1 [48.4–61.7]	0.041*
Sex (male)	12 (66.7%)	22 (62.9%)	0.777†
LVEF (%)	21.4 ± 5.8	23.9 ± 6.6	0.163*
HF aetiology — dilated	8 (44.4%)	16 (45.7%)	0.925†
HF aetiology — Chagas	6 (33.3%)	12 (34.3%)	0.943†
HF aetiology — ischaemic	2 (11.1%)	5 (14.3%)	0.731†
NT-proBNP (pg/mL)	6160 [4230–8880]	3662 [1380–6109]	0.030*
Initial dobutamine dose (µg/kg/min)	5.0 [5.0–5.0]	5.0 [5.0–5.0]	0.641*
Beta-blocker class — carvedilol	11 (61.1%)	22 (62.9%)	0.899†
Beta-blocker class — metoprolol	7 (38.9%)	13 (37.1%)	0.899†
Hypertension	8 (44.4%)	17 (48.6%)	0.763†
Diabetes mellitus	4 (22.2%)	9 (25.7%)	0.770†
CKD	3 (16.7%)	5 (14.3%)	0.809‡
AST (U/L)	38.0 [29.0–68.5]	29.0 [20.5–35.5]	0.028*
Albumin (g/dL)	3.4 [3.0–3.8]	3.5 [3.1–3.9]	0.412*
Creatinine (mg/dL)	1.4 [1.1–1.9]	1.3 [1.0–1.7]	0.521*
Sodium (mEq/L)	136 [133–139]	137 [134–140]	0.341*
**Panel B — Post-index clinical course**			
BB exposure duration after index (days)	58.5 [38.2–103.0]	144.0 [76.0–240.0]	0.007*
Hospitalisation duration (days)	86.0 [51.5–119.0]	150.0 [79.5–240.0]	0.037*
Final dobutamine dose (µg/kg/min)	20.0 [17.5–20.0]	13.2 [10.0–16.0]	< 0.001*
Mean dobutamine dose (µg/kg/min)	12.5 [12.0–13.3]	10.0 [8.5–11.5]	0.002*
ECMO initiation	0 (0.0%)	0 (0.0%)	N/A
Dialysis initiation	10 (55.6%)	1 (2.9%)	< 0.001‡
IABP insertion	15 (83.3%)	11 (31.4%)	0.001†
Other vasoactive agents	Milrinone: 7 (38.9%); Nitroprusside: 5 (27.8%)	Milrinone: 0 (0%); Nitroprusside: 4 (11.4%)	< 0.001†
Reason for BB suspension	Cardiogenic shock: 16 (88.9%); Septic shock: 2 (11.1%)	— (all maintained)	< 0.001†

*Mann–Whitney U test; †Pearson chi-square; ‡Fisher’s exact test. BB = beta-blocker; LVEF = left ventricular ejection fraction; HF = heart failure; NT-proBNP = N-terminal pro-brain natriuretic peptide; AST = aspartate aminotransferase; CKD = chronic kidney disease; ECMO = extracorporeal membrane oxygenation; IABP = intra-aortic balloon pump.

### Unadjusted outcomes

Although unadjusted comparisons demonstrate clinical differences between groups, these associations are descriptive only. Because death precludes transplantation, formal inference required competing-risk methods.

### Propensity weighting and covariate balance

IPTW improved covariate balance across the pre-specified baseline covariates included in the propensity score model. After weighting, 9 of 11 variables (81.8%) achieved an absolute SMD below 0.10. Residual imbalance persisted for LVEF (SMD = 0.28; lower in the BB-suspended group) and NT-proBNP (SMD = 0.47; higher in the BB-suspended group), both in the direction of greater disease severity in the BB-suspended group. Given the modest sample size, some residual imbalance in markers of disease severity cannot be fully excluded; estimates should therefore be interpreted as reflecting both a possible prognostic association and the prognostic advantage conferred by haemodynamic stability itself (Fig 1).

### Weighted competing-risk outcomes

**Fig 2:**
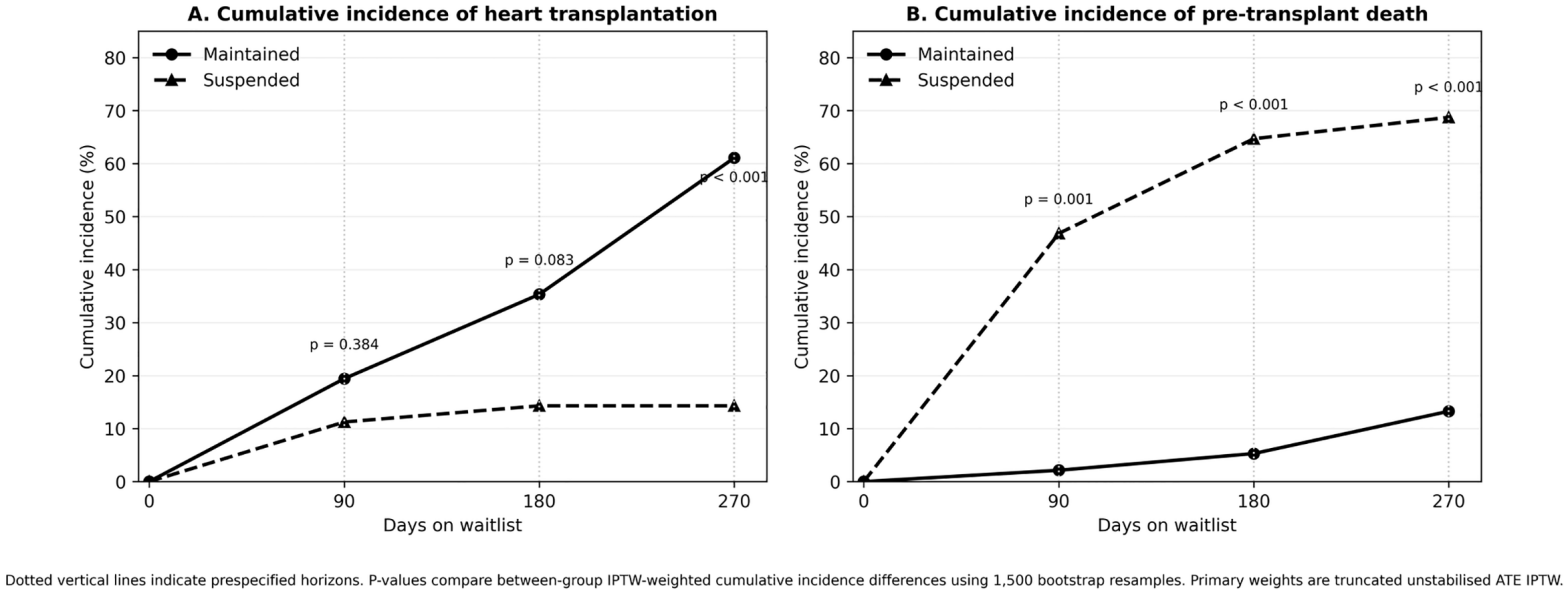
IPTW-weighted Aalen–Johansen cumulative incidence functions for heart transplantation (Panel A) and pre-transplant death (Panel B). Curves compare patients in whom beta-blockers were maintained (solid lines, n = 35) versus suspended (dashed lines, n = 18). Pre-transplant death was treated as a competing event for heart transplantation, and vice versa. Dashed vertical lines indicate pre-specified horizons at 90, 180, and ≥ 270 days. P-values correspond to between-group differences in IPTW-weighted cumulative incidence at each horizon derived from 1,500 bootstrap resamples. Primary weights were truncated unstabilised ATE IPTW. Abbreviations: IPTW, inverse probability of treatment weighting; ATE, average treatment effect; CIF, cumulative incidence function.

In IPTW-adjusted Aalen–Johansen analyses (Fig 2 and Table 2), BB continuation was associated with substantially higher cumulative incidence of HT and lower pre-transplant mortality:

**Table 2:** IPTW-weighted cumulative incidence of heart transplantation and pre-transplant death at pre-specified time horizons.

Endpoint	Days	BB Maintained (CIF, %)	BB Suspended (CIF, %)	Difference (pp)	95% CI (pp)	p-value
Heart transplant	90	19.4	11.2	+8.2	−12.5 to +26.3	0.384
Heart transplant	180	35.3	14.3	+21.0	−2.8 to +43.4	0.083
Heart transplant	≥ 270	61.1	14.3	+46.8	+21.8 to +70.6	< 0.001
Pre-Tx death	90	2.1	46.8	−44.7	−67.8 to −17.9	0.001
Pre-Tx death	180	5.3	64.7	−59.4	−83.3 to −30.2	< 0.001
Pre-Tx death	≥ 270	13.3	68.7	−55.5	−80.7 to −24.8	< 0.001

CIF = cumulative incidence function; IPTW = inverse probability of treatment weighting; pp = percentage points; Pre-Tx = pre-transplant. Primary outcome: HT at ≥ 270 days. 90-day and 180-day time points were pre-specified exploratory secondary horizons.

- Heart transplantation at ≥ 270 days: 61.1% vs 14.3% (absolute difference +46.8 percentage points [pp]; p < 0.001)
- Pre-transplant death at ≥ 270 days: 13.3% vs 68.7% (absolute difference −55.5 pp; p < 0.001)

**Fig 3:**
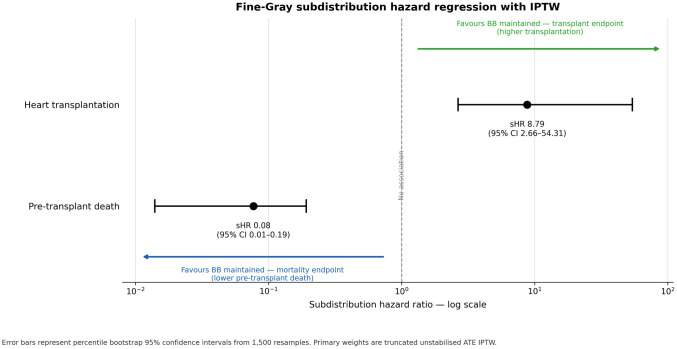
IPTW-weighted Fine–Gray subdistribution hazard regression. Forest plot of subdistribution hazard ratios (sHRs) for heart transplantation and pre-transplant death comparing beta-blocker maintenance versus suspension. Error bars represent percentile bootstrap 95% confidence intervals from 1,500 resamples. Values to the left of 1 indicate a lower subdistribution hazard; values to the right of 1 indicate a higher subdistribution hazard. Directional annotations indicate the clinically favourable direction for each competing-risk endpoint. Primary model: truncated unstabilised ATE IPTW Fine–Gray regression. Abbreviations: IPTW, inverse probability of treatment weighting; sHR, subdistribution hazard ratio; CI, confidence interval; ATE, average treatment effect; BB, beta-blocker.

Fine–Gray regression confirmed these associations (Fig 3):

- Heart transplantation: sHR = 8.79 (95% bootstrap CI 2.66–54.31; 29 transplants, of which 4 occurred in the BB-suspended group)
- Pre-transplant death: sHR = 0.077 (95% bootstrap CI 0.014–0.192; 16 pre-transplant deaths)

### Sensitivity analyses

All four pre-specified sensitivity analyses yielded directionally consistent results (Supplementary Table S2): (i) unweighted Fine–Gray: sHR HT 4.87 (95% CI 1.77–29.92), sHR death 0.108 (0.001–0.277); (ii) multivariable Fine–Gray: sHR HT 5.14 (1.49–44.94), sHR death 0.059 (0.001–0.172); (iii) LASSO-penalised PS: sHR HT 5.00 (1.74–25.14), sHR death 0.107 (0.022–0.289); (iv) doubly robust Fine–Gray: sHR death 0.047 (0.001–0.136); the heart-transplantation estimate from this model is not reported, as it did not converge owing to sparse events (n = 4 transplants in the BB-suspended group). E-value analyses indicated that an unmeasured confounder would require a conservative E-value of at least 3.99 (pre-transplant mortality CI limit) and at least 2.65 (heart transplantation CI limit) to shift the confidence interval to the null (Supplementary Table S2, Panel B).

## Discussion

To our knowledge, this is the first study to specifically examine outcomes associated with BB continuation during prolonged concomitant dobutamine infusion (≥ 30 consecutive days) in patients listed for heart transplantation. In this cohort, BB continuation was associated with a substantially higher probability of reaching transplantation and a lower probability of pre-transplant death, with associations consistent across four pre-specified sensitivity analyses. However, given the observational design and the specific clinical context in which BB suspension occurred, these findings should be interpreted as hypothesis-generating prognostic associations rather than evidence of a causal treatment effect.

The clinical scenario examined here — patients tolerating oral BB and continuous dobutamine for ≥ 30 days while awaiting transplantation — is poorly studied and increasingly relevant. As donor waiting times lengthen and access to durable mechanical circulatory support remains limited in many transplant programmes worldwide [19], prolonged pharmacological bridging with inotropes represents the primary or only available strategy for a substantial proportion of transplant candidates. Within this population, the present findings suggest that the sustained capacity to maintain BB therapy defines a clinically meaningful phenotype.

Prior studies examining BB–inotrope interactions have largely addressed acute scenarios: BB initiation during acute decompensation or short-term inotrope courses [7–10,17]. The target-trial emulation by Mori et al. reported neutral outcomes for BB *initiation* during dobutamine infusion in a broader acute HF population [17]. Our study addresses a fundamentally different question — whether to *continue* BB in patients who have already demonstrated tolerance to the combination over an extended period. Rather than being comparable to prior literature on initiation, our findings occupy an unstudied space and cannot be directly contrasted with or extrapolated from acute-setting data.

Competing-risk methodology was essential for this analysis. Because pre-transplant death directly precludes transplantation, Kaplan–Meier analyses would artificially inflate transplantation probabilities by censoring deaths [11–13]. The marked plateau in CIF for transplantation in the BB-suspended group after 90 days reflects the high competing mortality in that group — a pattern only visible, and correctly estimated, within a competing-risk framework. Competing-risk methods provide a more appropriate representation of waiting-list outcomes, particularly in populations with substantial short-term mortality risk such as patients receiving continuous inotropes or mechanical circulatory support [1,14,15].

The early divergence in pre-transplant mortality — 46.8% in the BB-suspended group versus 2.1% in the BB-maintained group by day 90 (p = 0.001) — is striking and requires careful interpretation. This pattern reflects two non-mutually exclusive phenomena: the direct haemodynamic consequences of BB suspension in a fragile population — potentially accelerating sympathetic reactivation and destabilisation — and the fact that BB suspension occurred exclusively in the context of already-established cardiogenic or septic shock, which itself carries high short-term mortality independent of any treatment decision. The retrospective design does not allow separation of these effects.

The observed association between BB continuation and improved waiting-list outcomes is biologically plausible and consistent with known pathophysiological mechanisms. BB therapy attenuates sympathetic overactivation, reduces arrhythmic burden, and may promote β-receptor resensitisation during sustained catecholaminergic stimulation [20–22]. These mechanisms could contribute to enhanced haemodynamic stability and maintenance of transplant eligibility in selected patients. Previous observational studies have reported favourable outcomes with BB therapy in advanced HF populations, although most were conducted outside the specific context of prolonged continuous dobutamine infusion [23–25].

After IPTW, 9 of 11 variables (81.8%) achieved adequate balance. Residual imbalance persisted for LVEF (SMD = 0.28; lower in the BB-suspended group) and NT-proBNP (SMD = 0.47; higher in the BB-suspended group), both in the direction of greater disease severity in the suspended group. Residual imbalance in both variables favoured the BB-maintained group and may partially explain the magnitude of the observed associations.

Confounding by indication is therefore the central interpretive caveat and is likely, as BB discontinuation occurred exclusively in the context of cardiogenic or septic shock. It is plausible that a substantial proportion of the observed association reflects the prognostic advantage of haemodynamic stability *per se*, rather than a direct pharmacological consequence of BB continuation. Patients who tolerated BB throughout follow-up may represent a selected subgroup with inherently better cardiovascular reserve. Accordingly, BB continuation should be interpreted primarily as a marker of relative haemodynamic stability — a phenotype with higher probability of reaching transplantation — rather than as a direct cause of improved outcomes. These findings do not support definitive causal inference. E-value analyses indicate that an unmeasured confounder would require a conservative E-value of at least 3.99 to explain away the mortality finding, substantially exceeding the magnitude of known prognostic markers after the adjustments applied, though unmeasured confounding cannot be excluded [18].

This reframing has direct clinical implications. In transplant centres that do not have access to durable ventricular assist devices, the present findings are most directly representative: identifying candidates who demonstrate sustained tolerance to combined BB and dobutamine therapy may serve as an actionable prognostic marker, and systematic monitoring to preserve this phenotype — through careful dose titration and early recognition of deterioration — may be a practical and cost-effective strategy. In centres with routine VAD availability, the clinical question itself changes, as mechanical bridging provides an alternative management pathway; whether the present findings translate to that context remains to be established.

### Limitations

Several limitations must be acknowledged. The retrospective, single-centre design limits generalisability. With only 18 patients in the smaller treatment group and eight model parameters, the events-per-parameter ratio was approximately 2.3 (3.0 per clinical covariate), well below the conventional threshold of 10 and constraining covariate inclusion [26]. The wide bootstrap CI for the transplantation sHR (2.66–54.31) reflects sparse events in the BB-suspended group (n = 4 transplants) and precludes precise point estimation; the CIF-based absolute differences are more stable and represent the primary evidence.

The 30-day qualifying period constitutes a form of symmetric left truncation: patients who died or reached transplantation before completing 30 days of combined therapy were excluded. Importantly, this truncation was identical across both exposure groups — all 53 patients entered the study at the same index date having completed the same qualifying period. This design eliminates the asymmetric immortal time bias that would arise if qualifying periods differed between groups, and it is intentional: the 30-day period defines the poorly studied clinical phenomenon of interest — prolonged sustained concomitant use — rather than representing a methodological limitation.

Confounding by indication cannot be fully excluded. The binary exposure classification does not capture subsequent dose modifications or transient interruptions, introducing potential time-varying misclassification. A marginal structural model with time-updated IPTW would provide a more rigorous causal estimate but requires substantially larger samples [16]. The inability to externally validate the findings underscores the need for multicentre prospective evaluation.

Given these limitations, the present findings should be considered hypothesis-generating and should not change clinical practice independently. The directional consistency across four sensitivity analyses and the E-value assessment support the credibility of the associations, but prospective validation is required.

Future prospective multicentre registries are needed to confirm these observations, standardise the definition of prolonged concomitant use, refine patient selection, and clarify the mechanistic interaction between chronic beta-blockade and prolonged inotrope dependence in the transplant setting.

## Conclusion

This study evaluates outcomes in patients sustaining oral beta-blocker therapy during prolonged dobutamine infusion while awaiting heart transplantation, a clinical scenario with limited available data. BB continuation was associated with a higher probability of transplantation, with consistent direction across pre-specified sensitivity analyses. Given the small sample size and the potential for residual confounding by indication, these findings should be interpreted with caution and are best viewed as hypothesis-generating. They may help inform future research, particularly prospective studies in this setting.

## Supporting information

S1 Text Propensity score distribution by treatment group. (DOCX)

S1 Table Sensitivity analyses and e-value assessment. (DOCX)

S1 File STROBE statement — checklist for cohort studies. (DOCX)

S1 Fig Supplementary figure. (TIF)
