# Supplementary material for: Continuation of beta-blockers during prolonged dobutamine infusion in heart transplant–prioritised patients: A competing-risk analysis
Source: PLoS One. 2026 Jul 21;21(7):e0354128. doi: 10.1371/journal.pone.0354128 (PMC13387565; doi:10.1371/journal.pone.0354128)
Supplement: S1 Text — (DOCX) [file pone.0354128.s001.docx]

**S1 Fig.** Propensity Score Distribution by Treatment Group.

The propensity score (PS) represents the estimated probability of BB maintenance for each patient, derived from a logistic regression model with 6 clinical covariates (encoded as 8 model parameters): age, sex, HF aetiology (3 dummy variables: Chagas, ischaemic, dilated), hypertension, diabetes mellitus, and BB class. All covariates were selected a priori based on clinical knowledge; post-index variables were excluded as mediators (VanderWeele 2009 [Reference 24]).

**Panel A:** Overlapping histograms of PS distribution for BB-maintained (blue, n = 35) and BB-suspended (red, n = 18) patients. Shaded green region indicates the common support area [0.338–0.858], representing the PS range in which patients from both groups are represented and over which IPTW is valid.

**Panel B:** Mirrored histogram facilitating visual comparison of distributional symmetry.

**Panel C:** Box-and-jitter plot with individual patient PS values overlaid. The diamond (◆) indicates the group mean. Boxes represent the interquartile range; the horizontal line indicates the median.

| **Parameter** | **Value** |
| --- | --- |
| **Working cohort** | N = 53 |
| BB maintained | n = 35 |
| BB suspended | n = 18 |
| PS range — BB maintained | 0.338–0.930 (mean 0.716; SD 0.164) |
| PS range — BB suspended | 0.185–0.858 (mean 0.552; SD 0.200) |
| PS median — maintained / suspended | 0.728 / 0.581 |
| Common support region | 0.338–0.858 |
| Patients within common support | n = 39 (73.6%) |
| **Unstabilised ATE IPTW weights (truncated 1st–99th)** | Range 1.080–6.153 |
| **Stabilised ATE IPTW weights (sensitivity)** | Range 0.451–2.164; materially similar results |
| **SMD after IPTW** | 9 of 11 variables (81.8%) achieved \|SMD\| < 0.10. Residual imbalance: LVEF (SMD = 0.28) and NT-proBNP (SMD = 0.47), both in the direction of greater disease severity in the BB-suspended group. |
| PS model | Logistic regression; unpenalised; 6 clinical covariates (8 model parameters); events-per-parameter ≈ 2.3 (3.0 per clinical covariate) |

*Abbreviations: PS, propensity score; BB, beta-blocker; HF, heart failure; IPTW, inverse probability of treatment weighting; ATE, average treatment effect; SMD, standardised mean difference; SD, standard deviation; LVEF, left ventricular ejection fraction; NT-proBNP, N-terminal pro-brain natriuretic peptide. Events-per-parameter calculated as n (smaller group) / model parameters = 18 / 8 ≈ 2.3; equivalently 18 / 6 = 3.0 per clinical covariate. All values from dataset_recoded.xlsx (N = 53).*
