# Supplementary material for: Continuation of beta-blockers during prolonged dobutamine infusion in heart transplant–prioritised patients: A competing-risk analysis
Source: PLoS One. 2026 Jul 21;21(7):e0354128. doi: 10.1371/journal.pone.0354128 (PMC13387565; doi:10.1371/journal.pone.0354128)
Supplement: S1 Table — (DOCX) [file pone.0354128.s002.docx]

**S1 Table.** Sensitivity Analyses and E-value Assessment.

*N = 53 (maintained = 35; suspended = 18). Primary model = IPTW-weighted Fine–Gray. Sensitivity analyses were pre-specified. All CIs from percentile-based nonparametric bootstrap (1,500 replications). Primary weights: truncated unstabilised ATE IPTW (range 1.080–6.153). Source: dataset_recoded.xlsx.*

**Panel A — Subdistribution Hazard Ratios Across Analytical Models**

| **Model** | **Analytical Model** | **sHR HT** | **95% CI** | **p** | **sHR Death** | **95% CI** | **p** |
| --- | --- | --- | --- | --- | --- | --- | --- |
| **Primary** | **IPTW Fine–Gray (6-covariate logistic PS; truncated unstabilised ATE IPTW)** | **8.79** | **2.66–54.31** | **<0.001** | **0.077** | **0.014–0.192** | **<0.001** |
| S1 | Unweighted Fine–Gray (no IPTW) | 4.87 | 1.77–29.92 | 0.001 | 0.108 | 0.001–0.277 | 0.001 |
| S2 | Multivariable Fine–Gray (age, LVEF¹, HF aetiology; no IPTW) | 5.14 | 1.49–44.94 | 0.001 | 0.059 | 0.001–0.172 | 0.001 |
| S3 | LASSO-penalised PS (cross-validated λ) | 5.00 | 1.74–25.14 | 0.003 | 0.107 | 0.022–0.289 | 0.003 |
| S4 | Doubly robust Fine–Gray (IPTW + covariate adjustment) | NR² | — | — | 0.047 | 0.001–0.136 | <0.001 |

*¹LVEF included in Sensitivity 2 as a key severity marker with clinically meaningful between-group difference (mean 21.4% vs 23.9%; p = 0.163) and residual post-IPTW imbalance (SMD = 0.28). ²Sensitivity 4: the doubly-robust heart-transplantation model did not converge owing to sparse events (n = 4 transplants in the suspended group) and is not reported (NR); the death estimate converged and is shown. Bold row = primary results. sHR, subdistribution hazard ratio; CI, 95% bootstrap CI (1,500 replications, percentile method); IPTW, inverse probability of treatment weighting; PS, propensity score; LVEF, left ventricular ejection fraction; HF, heart failure; LASSO, least absolute shrinkage and selection operator.*

**Panel B — E-value Analysis: Robustness to Unmeasured Confounding**

E-values quantify the minimum strength of association an unmeasured confounder would need with both the exposure (BB maintenance) and the outcome to fully explain away the observed association after all measured adjustments. The conservative correction for common outcomes is applied throughout (Ding & VanderWeele, Epidemiology 2016 [Reference 25]).

| **Model — Outcome** | **sHR** | **CI bound** | **E-value (point) cons.** | **E-value (CI limit) cons.** | **E-value (point) std.** | **Note** |
| --- | --- | --- | --- | --- | --- | --- |
| **Heart Transplantation** | | | | | | |
| **Primary IPTW — HT** | **8.79** | **2.66 (lo)** | **5.38** | **2.65** | **17.06** |  |
| Sensitivity 1 — HT | 4.87 | 1.77 (lo) | 3.84 | 1.99 | 9.21 |  |
| Sensitivity 2 — HT | 5.14 | 1.49 (lo) | 3.96 | 1.74 | 9.75 |  |
| Sensitivity 3 — HT | 5.00 | 1.74 (lo) | 3.90 | 1.97 | 9.47 |  |
| **Pre-transplant Death** | | | | | | |
| **Primary IPTW — Death** | **0.077** | **0.192 (hi)** | **6.67** | **3.99** | **25.46** |  |
| Sensitivity 1 — Death | 0.108 | 0.277 (hi) | 5.54 | 3.21 | 18.00 |  |
| Sensitivity 2 — Death | 0.059 | 0.172 (hi) | 7.70 | 4.26 | 33.39 |  |
| Sensitivity 3 — Death | 0.107 | 0.289 (hi) | 5.56 | 3.13 | 18.18 |  |
| Sensitivity 4 — Death | 0.047 | 0.136 (hi) | 8.69 | 4.87 | 42.05 |  |

*E-values were computed with the conservative correction for common outcomes (Ding & VanderWeele, Epidemiology 2016 [Reference 25]): the standard E-value formula was applied to √sHR (and to 1/sHR for the death outcome). Conservative E-values are reported at the point estimate and at the CI limit closest to the null; standard rare-outcome E-values (VanderWeele & Ding, Ann Intern Med 2017) are shown for reference only. lo, lower CI bound; hi, upper CI bound; cons., conservative correction; std., standard (rare-outcome) formula.*

**Panel C — Events-per-Variable Analysis**

The events-per-parameter ratio (events in the smaller group ÷ estimated model parameters) was 18 / 8 ≈ 2.3; the events-per-clinical-covariate ratio was 18 / 6 = 3.0. Both fall well below the conventional minimum of 10 (Peduzzi et al., J Clin Epidemiol 1996 [Reference 26]), justifying the parsimonious specification. HF aetiology was counted as 1 clinical covariate but encoded as 3 binary dummy variables (total model parameters = 8).

| **PS Specification** | **Clinical covariates** | **Model parameters** | **n smaller group** | **Events / parameter** |
| --- | --- | --- | --- | --- |
| **Primary: age, sex, HF aetiology (Chagas/ischaemic/dilated), hypertension, diabetes, BB class** | **6** | **8** | **18** | **2.3** |
| Hypothetical: Primary + LVEF, NT-proBNP, dialysis, IABP | 10 | 12 | 18 | 1.5 |
| Sensitivity 3: LASSO-penalised PS (cross-validated λ, auto-selection) | Auto | Auto | 18 | Optimised |

*Hypothetical model not used: the events-per-parameter ratio falls further below the minimum threshold and it includes post-index mediators. LASSO automatically handles sample-size constraints; results directionally consistent (Panel A). Reference: Peduzzi P et al. J Clin Epidemiol. 1996;49:1373–1379.*
