# Supplementary material for: Continuation of beta-blockers during prolonged dobutamine infusion in heart transplant–prioritised patients: A competing-risk analysis
Source: PLoS One. 2026 Jul 21;21(7):e0354128. doi: 10.1371/journal.pone.0354128 (PMC13387565; doi:10.1371/journal.pone.0354128)
Supplement: S1 File — (DOCX) [file pone.0354128.s003.docx]

**S1 File.** STROBE Statement — Checklist for Cohort Studies.

*Strengthening the Reporting of Observational Studies in Epidemiology.*

This is a retrospective single-centre cohort study. Items applicable only to prospective or randomised designs are marked N/A with justification. All manuscript locations refer to the revised manuscript submitted alongside this checklist.

| **STROBE Item** | **No.** | **Recommendation** | **Location in MS** | **Status** |
| --- | --- | --- | --- | --- |
| **TITLE AND ABSTRACT** | | | | |
| Title & abstract | 1a | Study design in title/abstract | Title; Abstract Methods | ✅ Yes |
|  | 1b | Informative balanced abstract | Abstract pp. 1–2 | ✅ Yes |
| **INTRODUCTION** | | | | |
| Background | 2 | Scientific background and rationale | Introduction pp. 3–4 | ✅ Yes |
| Objectives | 3 | Specific objectives and hypotheses | Introduction p. 4, final paragraph | ✅ Yes |
| **METHODS** | | | | |
| Study design | 4 | Key elements of study design at start of Methods | Methods → Study Design p. 5 | ✅ Yes |
| Setting | 5 | Setting, locations, relevant dates | Methods p. 5; Jan 2020–Dec 2023 | ✅ Yes |
| Participants | 6a | Eligibility criteria; follow-up description | Methods → Patient Selection pp. 5–6 | ✅ Yes |
|  | 6b | Matching criteria (if matched study) | Not applicable — IPTW used, no matching | N/A |
| Variables | 7 | Define outcomes, exposures, confounders | Methods pp. 5–7 | ✅ Yes |
| Data sources | 8 | Data sources and measurement methods | Methods → Data Collection p. 6 | ✅ Yes |
| Bias | 9 | Efforts to address bias | IPTW; 4 sensitivity analyses; E-values; mediator exclusion from PS | ✅ Yes |
| Study size | 10 | How study size arrived at | All eligible patients; no formal calculation | ✅ Yes |
| Quantitative vars | 11 | Handling of quantitative variables | Methods → Statistical Analysis p. 7 | ✅ Yes |
| Statistics | 12a | All statistical methods including confounding control | Methods pp. 6–7 | ✅ Yes |
|  | 12b | Subgroup/interaction analyses | Not pre-specified (N = 53) | N/A |
|  | 12c | Missing data | No missing data (stated in Methods) | ✅ Yes |
|  | 12d | Loss to follow-up | All censored at study end; no loss | ✅ Yes |
|  | 12e | Sensitivity analyses | Methods p. 7; results in S1 Table | ✅ Yes |
| **RESULTS** | | | | |
| Participants | 13a | Numbers at each stage (screened, eligible, included) | Results → Study Population p. 8 | ✅ Yes |
|  | 13b | Reasons for non-participation at each stage | Results p. 8: 30 exclusions with reasons | ✅ Yes |
|  | 13c | Flow diagram | Flow described in text; participant flow diagram recommended as S2 Fig | ⚠ Partial |
| Descriptive | 14a | Participant characteristics including exposures | Results + Table 1 pp. 8–9 | ✅ Yes |
|  | 14b | Missing data per variable | Methods: no missing data | ✅ Yes |
|  | 14c | Summarise follow-up time | Results: median hospitalisation 150 vs 86 days | ✅ Yes |
| Outcomes | 15 | Outcome events over time | Table 2; HT = 29, Death = 16, Censored = 8 | ✅ Yes |
| Main results | 16a | Unadjusted and adjusted estimates with precision | Table 2: IPTW CIFs + 95% CIs; sHRs with bootstrap CI | ✅ Yes |
|  | 16b | Category boundaries for categorised continuous variables | No continuous variables categorised | N/A |
|  | 16c | Translate to absolute risk | Table 2: absolute differences in percentage points | ✅ Yes |
| Other analyses | 17 | Sensitivity and other analyses | Results p. 10; S1 Table | ✅ Yes |
| **DISCUSSION** | | | | |
| Key results | 18 | Summary with reference to study objectives | Discussion p. 11, first paragraph | ✅ Yes |
| Limitations | 19 | Limitations including sources of bias with direction | Limitations pp. 14–15 | ✅ Yes |
| Interpretation | 20 | Cautious interpretation | Discussion + Conclusion pp. 14–16 | ✅ Yes |
| Generalisability | 21 | External validity | Discussion p. 13: resource-limited vs high-resource settings | ✅ Yes |
| **OTHER INFORMATION** | | | | |
| Funding | 22 | Source of funding and role of funders | Funding Statement p. 2: no specific funding | ✅ Yes |

*N/A, not applicable to retrospective cohort design. MS, manuscript; HT, heart transplantation; IPTW, inverse probability of treatment weighting; CIF, cumulative incidence function; PS, propensity score; sHR, subdistribution hazard ratio. Reference: von Elm E et al. STROBE Initiative. Lancet. 2007;370:1453–1457.*
