# Supplementary material for: Continuation of beta-blockers during prolonged dobutamine infusion in heart transplant–prioritised patients: A competing-risk analysis
Source: PLoS One. 2026 Jul 21;21(7):e0354128. doi: 10.1371/journal.pone.0354128 (PMC13387565; doi:10.1371/journal.pone.0354128)
Supplement: Attachment — Submitted filename: Response_to_Reviewers_PONE-D-25-62240_2026-06-23 (3).docx [file pone.0354128.s006.docx]

**Response to Reviewers**

*Manuscript PONE-D-25-62240 — “Continuation of Beta-Blockers During Prolonged Dobutamine Infusion in Heart Transplant–Prioritised Patients: A Competing-Risk Analysis”*

*Submission date: 23 June 2026*

We thank the Academic Editor and both reviewers for their careful and constructive evaluation of our manuscript. We have addressed every point raised; our responses are set out below, and all corresponding changes have been incorporated into the revised manuscript (changes are also marked in the tracked-changes version). Reviewer comments are reproduced verbatim; quoted excerpts in the right-hand column are taken from the revised manuscript.

| **Reviewer Comment** | **Authors’ Response** | **Changes in the Manuscript** |
| --- | --- | --- |
| **Academic Editor – Journal Requirements** | | |
| 1. Please ensure that your manuscript meets PLOS ONE’s style requirements, including those for file naming. | We thank the Academic Editor for these instructions. The manuscript, figures, and Supporting Information have been reformatted to conform to the PLOS ONE style templates, including the file-naming conventions for the title page, main body, and supplementary files. | **Location: Throughout (formatting only; no change to scientific content).**  Title page, section headings, table and figure captions, and Supporting Information file names aligned to the PLOS ONE templates. |
| 2. Please confirm whether your submission contains all raw data required to replicate the results, or deposit the data and provide URLs/DOIs; if restrictions apply, explain them and provide a data-access contact. | We confirm that the data underlying all reported findings are contained within the manuscript, its tables and figures, and the accompanying Supporting Information. Owing to Brazilian data-protection legislation (LGPD) and the non-negligible re-identification risk inherent to a 53-patient cohort, the individual-level dataset cannot be deposited in a public repository; however, a fully de-identified minimal dataset is available to qualifying researchers on application to the Institutional Ethics Committee, whose contact details are provided. The Data Availability Statement has been revised accordingly. | **Location: Data Availability Statement.**  *“Due to Brazilian data protection regulations (Lei Geral de Proteção de Dados – LGPD), individual-level clinical data cannot be publicly shared; the small sample size (N = 53) creates a non-negligible risk of patient re-identification. All aggregate data necessary to replicate the reported findings are presented within the manuscript and its Supporting Information files. Researchers meeting criteria for access to confidential data may request the de-identified dataset through the Ethics Committee of the Instituto Dante Pazzanese de Cardiologia (cep@idpc.org.br; CAAE 77180624.6.0000.5462).”* |
| 3. Your ethics statement should only appear in the Methods section. If it appears in any other section, please delete it from there. | We are grateful for this clarification. The ethics statement that previously appeared as a standalone section preceding the Introduction has been deleted, and the ethics information now appears exclusively within the Methods, under the sub-heading ‘Ethics Approval’. | **Location: Front matter — standalone ‘Ethics’ block deleted.**  **Location: Methods → Ethics Approval (retained).**  *“This retrospective study was approved by the Institutional Ethics Committee of the Instituto Dante Pazzanese de Cardiologia (CAAE 77180624.6.0000.5462) … All procedures complied with the Declaration of Helsinki.”* |
| 4. Please include a copy of Tables 2 and 3 which you refer to in your text on page 12. | We apologise for this oversight. The manuscript previously contained an erroneous citation to a non-existent ‘Table 3’. There is a single cumulative-incidence table, now correctly numbered Table 2; the in-text citation has been corrected and the table is presented in full within the Results. | **Location: Results → Weighted Competing-Risk Outcomes.**  *“In IPTW-adjusted Aalen–Johansen analyses (Figure 2 and Table 2) …”*  **Location: Table 2 included in full (cumulative-incidence functions at 90, 180 and ≥270 days).** |
| 5. If the reviewer comments recommend citing specific previously published works, please evaluate whether they are relevant. There is no requirement to cite unless indicated by the editor. | The reviewers did not recommend any specific works for citation. In support of the additional analyses introduced during revision, we have nonetheless added three methodological references that underpin the new methods. | **Location: References 24–26 (new).**  VanderWeele 2009 (consistency / mediator adjustment; Reference 24); Ding & VanderWeele 2016 (E-values; Reference 25); Peduzzi et al. 1996 (events-per-variable; Reference 26). |
| **Reviewer #1** | | |
| General assessment: “Thank you for the opportunity to review this thoughtful and clinically relevant manuscript … the use of competing-risk methodology is particularly appropriate and well executed … I support publication with minor revisions.” | We are grateful to Reviewer #1 for this encouraging assessment and for recognising the appropriateness of the competing-risk framework. Each of the suggestions is addressed below. | — |
| Confounding by indication. While the IPTW approach is appropriate, beta-blocker discontinuation appears closely linked to clinical instability (e.g., cardiogenic or septic shock). A brief additional discussion of how this may influence the observed associations would enhance the interpretation. | We agree entirely. The Discussion has been substantially expanded to position confounding by indication as the central interpretive caveat, making explicit that BB suspension occurred exclusively in the context of cardiogenic or septic shock and that the associations may, in substantial part, reflect the prognostic advantage of haemodynamic stability itself rather than a direct pharmacological effect. We have additionally quantified robustness to unmeasured confounding using E-values. | **Location: Discussion (confounding-by-indication paragraph) and Methods/Results (E-values).**  *“Confounding by indication is therefore the central interpretive caveat and is likely, as BB discontinuation occurred exclusively in the context of cardiogenic or septic shock … BB continuation should be interpreted primarily as a marker of relative haemodynamic stability … rather than as a direct cause of improved outcomes. These findings do not support definitive causal inference. E-value analyses indicate that an unmeasured confounder would require a conservative E-value of at least 3.99 to explain away the mortality finding …”* |
| Exposure definition and timing. Given the dynamic nature of beta-blocker management, a short comment on potential time-varying effects or misclassification would improve methodological transparency. | We thank the Reviewer. An explicit statement has been added to the Limitations acknowledging that the binary exposure definition does not capture subsequent dose modifications or transient interruptions and may therefore introduce time-varying misclassification; we note that a marginal structural model with time-updated weighting would be the appropriate design in a larger sample. | **Location: Limitations.**  *“The binary exposure classification does not capture subsequent dose modifications or transient interruptions, introducing potential time-varying misclassification. A marginal structural model with time-updated IPTW would provide a more rigorous causal estimate but requires substantially larger samples.”* |
| Generalizability. It would be helpful to briefly expand on how these findings may translate to other practice environments, including higher-resource centers. | We agree this warranted expansion. The Discussion now distinguishes explicitly between centres lacking durable mechanical circulatory support — to which our findings are most directly applicable — and higher-resource centres with routine VAD availability, in which the clinical question itself changes and the applicability of our findings remains to be established. | **Location: Discussion (clinical-implications paragraph).**  *“In transplant centres that do not have access to durable ventricular assist devices, the present findings are most directly representative … In centres with routine VAD availability, the clinical question itself changes, as mechanical bridging provides an alternative management pathway; whether the present findings translate to that context remains to be established.”* |
| Clarity and readability. A few sentences, particularly in the Introduction and Discussion, could be slightly streamlined to improve flow. | We thank the Reviewer. The Introduction and Discussion have been streamlined, redundant passages removed, and the rationale refocused on the specific, under-studied scenario of prolonged concomitant therapy. | **Location: Introduction (new framing paragraph); Discussion (streamlined throughout).**  *“However, a specific and underexplored clinical scenario exists: patients who have already been receiving oral BB and continuous dobutamine concomitantly for ≥30 consecutive days while awaiting transplantation … These patients have, by definition, demonstrated sustained haemodynamic tolerance to the combination …”* |
| **Reviewer #2 – Major Points** | | |
| General assessment: the clinical question, the cohort, and the analytic framework all have real value … “The issues lie in the implementation. Addressing them will require engaging more directly with the confounding by indication, clarifying how the sensitivity analyses are reported, and reframing the findings as hypothesis-generating rather than causal.” | We are grateful to Reviewer #2 for a rigorous and constructive appraisal that has materially improved the manuscript. We have engaged directly with confounding by indication, clarified the reporting of the sensitivity analyses, and reframed the findings as hypothesis-generating throughout, as detailed below. | — |
| The propensity model includes only age, sex, HF etiology, hypertension, diabetes, and BB class, and omits the variables that drove the treatment decision: cardiogenic shock, dobutamine dose, NT-proBNP, IABP, and dialysis … The authors should display the propensity score distributions in each group to show overlap … Confounding by indication should be discussed directly as the leading alternative explanation. | We appreciate this important methodological point and wish to clarify a key issue of temporality. On chart review, the variables identified by the Reviewer — cardiogenic shock, dobutamine dose escalation, IABP, and dialysis — were confirmed to have occurred after the index date; they are therefore post-baseline mediators of haemodynamic deterioration rather than baseline confounders, and their inclusion in the propensity score would have introduced mediator-adjustment bias. We now state this explicitly, present baseline (Panel A) and post-index (Panel B) variables separately in Table 1, justify the parsimonious six-covariate model on events-per-parameter grounds, display the propensity-score distributions by group (Supplementary Figure S1), and discuss confounding by indication directly as the leading alternative explanation.  We further clarify the treatment of LVEF and NT-proBNP, which the Reviewer specifically noted. These were deliberately reserved for supplementary severity assessment rather than entered into the primary propensity score, for three reasons: (i) parsimony — with only 18 patients in the smaller group, adding further covariates would have reduced the events-per-parameter ratio still further and risked overfitting and unstable propensity-score estimation; (ii) both were considered markers of baseline disease severity and were therefore evaluated through covariate-balance diagnostics and sensitivity analyses rather than included in the primary treatment-assignment model; and (iii) neither was ignored — both were displayed transparently in the Love plot (Figure 1), and LVEF was additionally incorporated into a pre-specified multivariable Fine–Gray sensitivity analysis. The consistent direction of the results across the weighted, multivariable, doubly robust, and penalised analyses indicates that the primary findings were unlikely to be driven solely by differences in baseline disease severity. | **Location: Methods → Statistical Analysis.**  *“Variables reflecting post-index haemodynamic deterioration — dialysis, IABP, dobutamine dose escalation, and shock episodes — were excluded because chart review confirmed these events occurred after the index date; their inclusion would constitute mediator adjustment bias. Furthermore, with only 18 patients in the smaller treatment group and eight estimated model parameters, the events-per-parameter ratio was low (≈ 2.3), well below the conventional minimum of 10, justifying a parsimonious specification.”*  **Location: Methods → Statistical Analysis; Table 1 (Panel A / Panel B); Supplementary Figure S1.**  *“Propensity score distributions by group are shown in Supplementary Figure S1.”*  **Location: Methods → Statistical Analysis (new sentence on LVEF / NT-proBNP).**  *“Accordingly, LVEF and NT-proBNP were considered baseline markers of disease severity and were therefore evaluated separately in covariate-balance diagnostics (Figure 1) and sensitivity analyses (LVEF in a pre-specified multivariable Fine–Gray model) rather than entered into the primary propensity score.”* |
| The transplant sHR of 8.69 (CI 1.90–75.90) and death sHR of 0.08 (CI 0.01–0.24) span nearly two orders of magnitude … only 16 deaths and 29 transplants across 53 patients … yet the Discussion characterises the findings as “robust” and “consistent” … The sensitivity analyses are not reported. Please soften the language, report the number of events and CI widths alongside the headline sHRs, report the sensitivity analyses as supplementary material, and acknowledge in the Limitations that the sample is too small for firm inference from the magnitude of the sHRs. | We agree and have made several changes. The descriptors ‘robust’ and ‘consistent’ have been removed where they implied certainty; the number of events is now reported alongside every headline sHR, with emphasis on the confidence-interval widths; the four pre-specified sensitivity analyses (unweighted, multivariable, LASSO-penalised, and doubly robust Fine–Gray) are now reported in full with their results and covariates (Supplementary Table S2); and the Limitations explicitly acknowledge that the sample is too small to support firm inference from the magnitude of the sHRs, identifying the more stable CIF-based absolute differences as the primary evidence. | **Location: Results → Weighted Competing-Risk Outcomes (events now reported).**  *“Heart transplantation: sHR = 8.79 (95% bootstrap CI 2.66–54.31; 29 transplants, of which 4 occurred in the BB-suspended group). Pre-transplant death: sHR = 0.077 (95% bootstrap CI 0.014–0.192; 16 pre-transplant deaths).”*  **Location: Results → Sensitivity Analyses; Supplementary Table S2 (Panel A).**  *“All four pre-specified sensitivity analyses yielded directionally consistent results (Supplementary Table S2): (i) unweighted Fine–Gray: sHR HT 4.87 (1.77–29.92), sHR death 0.108 (0.001–0.277); (ii) multivariable Fine–Gray: sHR HT 5.14 (1.49–44.94), sHR death 0.059 (0.001–0.172); (iii) LASSO-penalised PS: sHR HT 5.00 (1.74–25.14), sHR death 0.107 (0.022–0.289); (iv) doubly robust Fine–Gray: sHR death 0.047 (0.001–0.136); the heart-transplantation estimate from this model did not converge owing to sparse events and is not reported.”*  **Location: Limitations.**  *“The wide bootstrap CI for the transplantation sHR (2.66–54.31) reflects sparse events in the BB-suspended group (n = 4 transplants) and precludes precise point estimation; the CIF-based absolute differences are more stable and represent the primary evidence.”* |
| The pattern of results argues against a clean causal interpretation: the death difference is significant immediately (by day 90) while the transplant difference reaches significance only at ≥270 days … statements such as “novel evidence supporting” imply a stronger causal interpretation than the data support. The Discussion and Conclusion could be softened to associative phrasing, and the findings framed as hypothesis-generating. | We accept this entirely. The phrase ‘novel evidence supporting’ and other causal formulations have been removed and replaced with associative, hypothesis-generating language in the Abstract, Discussion, and Conclusion. A dedicated passage now interprets the early divergence in mortality as reflecting both the haemodynamic consequences of BB suspension and the fact that suspension occurred only in patients already in established shock, and we state that the retrospective design cannot separate these effects. | **Location: Discussion (early-divergence paragraph).**  *“The early divergence in pre-transplant mortality — 46.8% in the BB-suspended group versus 2.1% in the BB-maintained group by day 90 (p = 0.001) — is striking and requires careful interpretation … The retrospective design does not allow separation of these effects.”*  **Location: Conclusion (reframed).**  *“Given the small sample size and the potential for residual confounding by indication, these findings should be interpreted with caution and are best viewed as hypothesis-generating.”* |
| **Reviewer #2 – Minor Points** | | |
| The Abstract reports different cumulative incidence values at ≥270 days than the body (HT 61% vs 14%, p = 0.001 vs 68.2% vs 23.5%, p = 0.002 in Table 2; death 13% vs 69%, p < 0.001 vs 20.3% vs 63.7%, p = 0.012). Please reconcile. | We apologise for the discrepancy, which arose because the Abstract and Table reported different weighting schemes. The analysis has been re-specified to a single, consistently applied weighting approach (unstabilised truncated ATE-IPTW), and the cumulative-incidence values and p-values are now identical across the Abstract, the Results text, and Table 2. | **Location: Abstract and Table 2 (now concordant).**  *“Abstract: BB continuation was associated with a higher cumulative incidence of HT at ≥270 days (61.1% vs 14.3%; p < 0.001) and a lower cumulative incidence of pre-transplant death (13.3% vs 68.7%; p < 0.001) — identical to the ≥270-day row of Table 2.”* |
| The Methods describe ATE weights truncated at the 1st and 99th percentiles, while the Table 2 legend describes stabilized IPTW. Please reconcile to one consistent description. | We have reconciled the description. The Methods and the Table 2 legend now both specify unstabilised ATE weights truncated at the 1st and 99th percentiles as the primary approach; stabilised IPTW is reported only as a sensitivity analysis, where it yielded materially similar results. | **Location: Methods → Statistical Analysis.**  *“Unstabilised average treatment effect (ATE) weights were calculated as the inverse probability of the observed treatment assignment and truncated at the 1st and 99th percentiles … Stabilised IPTW weights were examined as a sensitivity analysis and yielded materially similar results (range 0.451–2.164).”*  **Location: Table 2 legend.**  *“Aalen–Johansen estimator with unstabilised ATE-IPTW (truncated 1st–99th percentile).”* |
| Methods contain an inconsistency in the exposure definition: inclusion requires ≥30 consecutive days of concomitant therapy with t=0 after that period, yet the BB-suspended group is defined as discontinuation ≈48–72 h before the index date. Please clarify and specify whether BB suspension occurred after the index date. | We thank the Reviewer for identifying this inconsistency. The exposure definition has been rewritten. By inclusion, both groups completed the 30-day concomitant period; the exposure (continuation versus permanent suspension) is now defined unambiguously as occurring ≥72 hours after the index date, with suspension occurring in the context of acute haemodynamic deterioration. | **Location: Methods → Exposure Definition.**  *“BB suspended: permanent discontinuation of oral BB therapy documented ≥72 hours after the index date, occurring in the context of acute haemodynamic deterioration (cardiogenic or septic shock), with no subsequent reinitiation at any point during follow-up.”* |
| Table 1: the percentages reported for the BB-suspended group correspond to a denominator of 19, while the stated group size is n = 18. Please reconcile. | Corrected. All proportions for the BB-suspended group are now calculated against the correct denominator of n = 18. | **Location: Table 1.**  Recalculated proportions, e.g.: Dialysis initiation 10 (55.6%); IABP insertion 15 (83.3%); Reason for BB suspension — Cardiogenic shock 16 (88.9%), Septic shock 2 (11.1%). |
| In Results “TGO” is undefined and should be replaced with AST (aspartate aminotransferase). | Corrected throughout. ‘TGO’ has been replaced with ‘AST (aspartate aminotransferase)’ in the Methods and Table 1. | **Location: Methods → Data Collection; Table 1.**  *“… laboratory data (sodium, creatinine, albumin, NT-proBNP, AST [aspartate aminotransferase]) … / Table 1: AST (U/L) 38.0 [29.0–68.5] vs 29.0 [20.5–35.5].”* |
| The Results refer to ‘Figure 2 and Table 3’, but no Table 3 is provided. | Corrected; the text now refers to ‘Figure 2 and Table 2’. No Table 3 exists. | **Location: Results → Weighted Competing-Risk Outcomes.**  *“In IPTW-adjusted Aalen–Johansen analyses (Figure 2 and Table 2) …”* |
| Discussion states “IPTW resulted in excellent covariate balance, with 96% of variables achieving an SMD < 0.10,” however Figure 1 shows 2 of 11 variables remain above threshold (≈82%). Please clarify the denominator and reconcile. | Reconciled. The Results and Discussion now state that 9 of 11 variables (81.8%) achieved \|SMD\| < 0.10 after weighting, consistent with Figure 1, and the over-stated ‘96%’ figure has been removed. | **Location: Results → Propensity Weighting and Covariate Balance; Discussion.**  *“After weighting, 9 of 11 variables (81.8%) achieved an absolute SMD below 0.10.”* |
| Discussion states the only variable with marginal residual imbalance was BB duration, but Figure 1 shows LVEF and NT-proBNP as the imbalanced variables, and BB duration does not appear on the balance plot. | Corrected. The text now correctly identifies LVEF (SMD = 0.28) and NT-proBNP (SMD = 0.47) as the variables with residual imbalance after weighting, consistent with Figure 1; the erroneous reference to ‘BB duration’ has been removed. | **Location: Results → Propensity Weighting and Covariate Balance; Discussion.**  *“Residual imbalance persisted for LVEF (SMD = 0.28; lower in the BB-suspended group) and NT-proBNP (SMD = 0.47; higher in the BB-suspended group), both in the direction of greater disease severity in the BB-suspended group.”* |
| Figure 3 truncates the upper bound of the heart-transplantation 95% CI (the CI extends to 75.90 but the x-axis ends at 50). Please extend the axis. | The x-axis of Figure 3 has been extended so that the full upper bound of the heart-transplantation confidence interval is displayed. Following re-specification of the primary weighting, the transplantation sHR is 8.79 (95% bootstrap CI 2.66–54.31), and the axis now encompasses this interval. | **Location: Figure 3 (revised) and legend.**  *“Forest plot of subdistribution hazard ratios (sHRs) … Error bars represent percentile bootstrap 95% confidence intervals from 1,500 resamples … (axis extended to display the full upper confidence limit).”* |
| Language: British and American spellings are used inconsistently (e.g., “aetiology” and “haemodynamic” alongside American spellings). Please apply one convention consistently. | British English has been applied consistently throughout the manuscript. | **Location: Throughout.**  Standardised spellings include: aetiology, ischaemic, haemodynamic, minimise, summarise, visualisation, utilisation, prioritised. |
| Please provide a completed STROBE checklist as supplementary material. | A completed STROBE checklist for cohort studies has been prepared and uploaded as Supporting Information. | **Location: Supporting Information (STROBE checklist, S File).**  Provided as a separate Supporting Information file accompanying the revised submission. |
