# Supplementary material for: Continuation of beta-blockers during prolonged dobutamine infusion in heart transplant–prioritised patients: A competing-risk analysis
Source: PLoS One. 2026 Jul 21;21(7):e0354128. doi: 10.1371/journal.pone.0354128 (PMC13387565; doi:10.1371/journal.pone.0354128)
Supplement: Peer review history

# Peer review: Decision Letter 0

**DOI:** 10.1371/journal.pone.0354128.r001

**Authors:** Moises Auron

7 Jun 2026

PONE-D-25-62240

Continuation of Beta-Blockers During Prolonged Dobutamine Infusion in Heart Transplant–Prioritised Patients: A Competing-Risk Analysis

PLOS One

Dear Dr. Rossi Neto,

Thank you for submitting your manuscript to PLOS ONE. After careful consideration, we feel that it has merit but does not fully meet PLOS ONE’s publication criteria as it currently stands. Therefore, we invite you to submit a revised version of the manuscript that addresses the points raised during the review process.

Please submit your revised manuscript by Jul 22 2026 11:59PM. If you will need more time than this to complete your revisions, please reply to this message or contact the journal office at plosone@plos.org. When you're ready to submit your revision, log on to https://www.editorialmanager.com/pone/ and select the 'Submissions Needing Revision' folder to locate your manuscript file.

Please include the following items when submitting your revised manuscript:

- A letter that responds to each point raised by the academic editor and reviewer(s). You should upload this letter as a separate file labeled 'Response to Reviewers'.
- A marked-up copy of your manuscript that highlights changes made to the original version. You should upload this as a separate file labeled 'Revised Manuscript with Track Changes'.
- An unmarked version of your revised paper without tracked changes. You should upload this as a separate file labeled 'Manuscript'.

If you would like to make changes to your financial disclosure, please include your updated statement in your cover letter. Guidelines for resubmitting your figure files are available below the reviewer comments at the end of this letter.

If applicable, we recommend that you deposit your laboratory protocols in protocols.io to enhance the reproducibility of your results. Protocols.io assigns your protocol its own identifier (DOI) so that it can be cited independently in the future. For instructions see: https://journals.plos.org/plosone/s/submission-guidelines#loc-laboratory-protocols. Additionally, PLOS ONE offers an option for publishing peer-reviewed Lab Protocol articles, which describe protocols hosted on protocols.io. Read more information on sharing protocols at https://plos.org/protocols?utm_medium=editorial-email&utm_source=authorletters&utm_campaign=protocols.

As the corresponding author, your ORCID iD is verified in the submission system and will appear in the published article. PLOS supports the use of ORCID, and we encourage all coauthors to register for an ORCID iD and use it as well. Please encourage your coauthors to verify their ORCID iD within the submission system before final acceptance, as unverified ORCID iDs will not appear in the published article. *Only*  the individual author can complete the verification step; PLOS staff *cannot*  verify ORCID iDs on behalf of authors.

We look forward to receiving your revised manuscript.

Kind regards,

Moises Auron, MD, FAAP, FACP, SFHM, FRCP (Lon), FRCPCH

Academic Editor

PLOS One

Journal Requirements:

When submitting your revision, we need you to address these additional requirements.

1. Please ensure that your manuscript meets PLOS ONE's style requirements, including those for file naming. The PLOS ONE style templates can be found at

https://journals.plos.org/plosone/s/file?id=wjVg/PLOSOne_formatting_sample_main_body.pdf and

https://journals.plos.org/plosone/s/file?id=ba62/PLOSOne_formatting_sample_title_authors_affiliations.pdf

2. We note that your Data Availability Statement is currently as follows: “All relevant data are within the manuscript and its Supporting Information files.”

Please confirm at this time whether or not your submission contains all raw data required to replicate the results of your study. Authors must share the “minimal data set” for their submission. PLOS defines the minimal data set to consist of the data required to replicate all study findings reported in the article, as well as related metadata and methods (https://journals.plos.org/plosone/s/data-availability#loc-minimal-data-set-definition).

For example, authors should submit the following data:

- The values behind the means, standard deviations and other measures reported;

- The values used to build graphs;

- The points extracted from images for analysis.

Authors do not need to submit their entire data set if only a portion of the data was used in the reported study.

If your submission does not contain these data, please either upload them as Supporting Information files or deposit them to a stable, public repository and provide us with the relevant URLs, DOIs, or accession numbers. For a list of recommended repositories, please see https://journals.plos.org/plosone/s/recommended-repositories.

If there are ethical or legal restrictions on sharing a de-identified data set, please explain them in detail (e.g., data contain potentially sensitive information, data are owned by a third-party organization, etc.) and who has imposed them (e.g., an ethics committee). Please also provide contact information for a data access committee, ethics committee, or other institutional body to which data requests may be sent. If data are owned by a third party, please indicate how others may request data access.

3. Your ethics statement should only appear in the Methods section of your manuscript. If your ethics statement is written in any section besides the Methods, please delete it from any other section.

4. Please include a copy of Tables 2 and 3 which you refer to in your text on page 12.

5. If the reviewer comments include a recommendation to cite specific previously published works, please review and evaluate these publications to determine whether they are relevant and should be cited. There is no requirement to cite these works unless the editor has indicated otherwise.

[Note: HTML markup is below. Please do not edit.]

Reviewers' comments:

Reviewer's Responses to Questions

**Comments to the Author**

1. Is the manuscript technically sound, and do the data support the conclusions?

The manuscript must describe a technically sound piece of scientific research with data that supports the conclusions. Experiments must have been conducted rigorously, with appropriate controls, replication, and sample sizes. The conclusions must be drawn appropriately based on the data presented.

Reviewer #1: Yes

Reviewer #2: Partly

**********

2. Has the statistical analysis been performed appropriately and rigorously?

Reviewer #1: Yes

Reviewer #2: No

**********

3. Have the authors made all data underlying the findings in their manuscript fully available?

The PLOS Data policy requires authors to make all data underlying the findings described in their manuscript fully available without restriction, with rare exception (please refer to the Data Availability Statement in the manuscript PDF file). The data should be provided as part of the manuscript or its supporting information, or deposited to a public repository. For example, in addition to summary statistics, the data points behind means, medians and variance measures should be available. If there are restrictions on publicly sharing data—e.g. participant privacy or use of data from a third party—those must be specified.

Reviewer #1: Yes

Reviewer #2: Yes

**********

4. Is the manuscript presented in an intelligible fashion and written in standard English?

PLOS ONE does not copyedit accepted manuscripts, so the language in submitted articles must be clear, correct, and unambiguous. Any typographical or grammatical errors should be corrected at revision, so please note any specific errors here.

Reviewer #1: Yes

Reviewer #2: Yes

**********

5. Review Comments to the Author

Please use the space provided to explain your answers to the questions above. You may also include additional comments for the author, including concerns about dual publication, research ethics, or publication ethics. (Please upload your review as an attachment if it exceeds 20,000 characters)

Reviewer #1: Thank you for the opportunity to review this thoughtful and clinically relevant manuscript. You address an important and understudied question in advanced heart failure, and the use of competing-risk methodology is particularly appropriate and well executed. The manuscript is clearly written, well structured, and the findings are both interesting and potentially practice-informing.

I have a few minor to moderate suggestions that may further strengthen the paper:

Confounding by indication

While the IPTW approach is appropriate, beta-blocker discontinuation appears closely linked to clinical instability (e.g., cardiogenic or septic shock). A brief additional discussion acknowledging how this may influence the observed associations would enhance the interpretation.

Exposure definition and timing

The exposure definition is clear and pragmatic. Given the dynamic nature of beta-blocker management in this population, a short comment on potential time-varying effects or misclassification would improve methodological transparency.

Generalizability

This study provides valuable real-world insights, particularly in settings with limited access to mechanical support. It would be helpful to briefly expand on how these findings may translate to other practice environments, including higher-resource centers.

Clarity and readability

The manuscript is overall very well written. A few sentences, particularly in the Introduction and Discussion, could be slightly streamlined to improve flow, but this is minor.

Overall, this is a strong and clinically meaningful study. I support publication with minor revisions.

Reviewer #2: The authors ask whether continuing beta-blocker (BB) therapy improves wait-list outcomes in heart transplant candidates who require prolonged dobutamine infusion. They report a retrospective single-center cohort study in São Paulo, Brazil, between January 2020 and December 2023, including 53 adults with advanced heart failure who received at least 30 consecutive days of concomitant BB and dobutamine therapy. Patients were classified after the index date as BB-maintained (n=35) or BB-suspended (n=18). The authors applied inverse probability of treatment weighting (IPTW) based on six baseline characteristics (age, sex, HF etiology, hypertension, diabetes, and BB class), analyzed outcomes with Aalen–Johansen cumulative incidence functions and Fine–Gray subdistribution hazard regression, and derived confidence intervals from 1,500 nonparametric bootstrap replicates. They report a markedly higher cumulative incidence of heart transplant (HT) in the BB-maintained group and lower pre-transplant mortality, and conclude that BB continuation may benefit selected transplant candidates.

The clinical question is genuinely important. BB management during prolonged inotrope dependence has no clear guideline, and the cohort reflects a setting that many transplant programs face, particularly those without ready access to durable mechanical circulatory support. The choice of methods is also reasonable: competing-risk analysis is the right framework for wait-list outcomes, and combining IPTW with Fine–Gray regression is a defensible approach to a non-randomized comparison. The clinical question, the cohort, and the analytic framework all have real value, and the paper could contribute meaningfully to the wait-list outcomes literature. The issues lie in the implementation. Addressing them will require engaging more directly with the confounding by indication, clarifying how the sensitivity analyses are reported, and reframing the findings as hypothesis-generating rather than causal.

The propensity model includes only age, sex, HF etiology, hypertension, diabetes, and BB class, and omits the variables that drove the treatment decision: cardiogenic shock (89.5% of suspended patients), dobutamine dose, NT-proBNP, IABP, and dialysis. The exposure (BB suspension) and the outcomes (death, non-transplant) therefore share a common upstream cause, clinical instability, and the analysis cannot separate a treatment effect from the prognostic effect of being stable enough to keep the BB on. The authors should display the propensity score distributions in each group to show the degree of overlap, recognizing that the propensity score model does not include the severity markers that drove treatment assignment. Confounding by indication should then be discussed directly in the Discussion and Limitations as the leading alternative explanation.

The transplant sHR of 8.69 indicates an eightfold higher rate of heart transplantation in the BB-maintained group, with a 95% confidence interval of 1.90 to 75.90. The death sHR of 0.08 indicates a comparably large reduction in pre-transplant mortality (CI 0.01 to 0.24). Both intervals span nearly two orders of magnitude, and effect sizes of this scale are outside what has been previously reported. The wide intervals may reflect how sparse the data are: only 16 deaths and 29 transplants across 53 patients. Despite this, the Discussion characterizes the findings as "robust" and "consistent." The sensitivity analyses described in the Methods, including a multivariable Fine–Gray model, are the natural way to test that claim, but their results are not reported. The authors should soften the language around the effect estimates throughout and report the number of events and confidence interval widths alongside the headline sHRs. The sensitivity analyses already performed, including the unweighted and multivariable Fine–Gray model results and the variables used in each, can be reported as supplementary material if available. The Limitations should acknowledge that the sample is too small to draw firm inferences from the magnitude of the sHRs.

The pattern of results argues against a clean causal interpretation: the death difference between groups is significant immediately (51.2% mortality in the suspended group vs 5.4% in the maintained group by day 90, p<0.001), while the transplant difference reaches significance at ≥270 days. The early divergence in death could be due to other variables, other than BBs, such as already-deteriorating patients dying soon after BB suspension. Given the retrospective single-center design, the sample size, and the unresolved confounding by indication, statements such as "novel evidence supporting" the practice imply a stronger causal interpretation than the data support. The Discussion and Conclusion could be softened to use more associative phrasing, and the findings might be more appropriately framed as hypothesis-generating.

Minor revisions:

• The Abstract reports different cumulative incidence values at ≥270 days than the body (HT 61% vs 14%, p = 0.001 in the Abstract vs 68.2% vs 23.5%, p = 0.002 in Table 2; pre-transplant death 13% vs 69%, p < 0.001 in the Abstract vs 20.3% vs 63.7%, p = 0.012 in Table 2). Please reconcile.

• The Methods describe ATE weights truncated at the 1st and 99th percentiles, while the Table 2 legend describes stabilized IPTW. These are different weighting procedures that may yield different estimates. The reader cannot tell which was used. Please reconcile the Methods and Table 2 legend to one consistent description of the weighting approach.

• Methods contain an inconsistency in the exposure definition.

Inclusion requires ≥30 consecutive days of concomitant BB and dobutamine therapy, with t=0 defined as the day following completion of that period. However, the BB-suspended group is defined as discontinuation of BB ≥48–72 hours before the index date with no reinitiation. This cannot be reconciled with the 30-day concomitant requirement. Please clarify the exposure definition and specify whether BB suspension actually occurred after the index date.

• Table 1 The percentages reported for the BB-suspended group correspond to a denominator of 19, while the stated group size is n = 18. Please reconcile this.

• In Results "TGO" is undefined and should be replaced with AST (aspartate aminotransferase).

• The Results section refers to 'Figure 2 and Table 3,' but no Table 3 is provided in the manuscript.

• Discussion states "IPTW resulted in excellent covariate balance, with 96% of variables achieving an SMD < 0.10," however, Figure 1 shows 2 of 11 variables remain above the threshold resulting in approximately 82% achieved balance. Please clarify the denominator used and reconcile the data.

• Discussion states that "the only variable with marginal residual imbalance was BB duration," but Figure 1 shows LVEF and NT-proBNP as the imbalanced variables after IPTW, and BB duration does not appear on the balance plot on the Y-axis.

• Figure 3 truncates the upper bound of the heart transplantation 95% CI (the CI extends to 75.90 but the x-axis ends at 50). Please extend the axis.

• Language British and American spellings are used inconsistently e.g., "aetiology" and "haemodynamic" appear alongside other American spellings. Please apply one convention consistently.

• Please provide a completed STROBE checklist as supplementary material.

**********

6. PLOS authors have the option to publish the peer review history of their article (what does this mean?). If published, this will include your full peer review and any attached files.

If you choose “no”, your identity will remain anonymous but your review may still be made public.

**Do you want your identity to be public for this peer review?**  For information about this choice, including consent withdrawal, please see our Privacy Policy.

Reviewer #1: No

Reviewer #2: No

**********

[NOTE: If reviewer comments were submitted as an attachment file, they will be attached to this email and accessible via the submission site. Please log into your account, locate the manuscript record, and check for the action link "View Attachments". If this link does not appear, there are no attachment files.]

To ensure your figures meet our technical requirements, please review our figure guidelines: https://journals.plos.org/plosone/s/figures

You may also use PLOS’s free figure tool, NAAS, to help you prepare publication quality figures: https://journals.plos.org/plosone/s/figures#loc-tools-for-figure-preparation.

NAAS will assess whether your figures meet our technical requirements by comparing each figure against our figure specifications.

# Author comment: Author response to Decision Letter 1

**DOI:** 10.1371/journal.pone.0354128.r002

25 Jun 2026

We thank the Academic Editor and both Reviewers for their thorough and constructive evaluation. We have addressed every comment raised; our detailed point-by-point responses are provided in the accompanying "Response to Reviewers" file uploaded with this submission.

In summary:

All statistical, numerical, and terminology inconsistencies identified by both reviewers have been corrected (Abstract/Table 2 concordance, covariate balance percentages, imbalanced variable identification, Table 1 denominators, exposure definition temporality, Figure 3 axis extension).

Confounding by indication is now explicitly positioned as the central interpretive caveat, with E-value analyses added (Supplementary Table S1, Panel B).

Sensitivity analyses are reported in full (Supplementary Table S1, Panel A).

The propensity score model specification is justified on grounds of mediator exclusion and events-per-parameter constraints, with propensity score distributions displayed (S1 Fig).

All causal language has been replaced with associative, hypothesis-generating framing throughout.

PLOS ONE formatting requirements have been fully implemented (Author Contributions, Acknowledgements, Keywords placement, figure legends, table footnotes, reference renumbering, TIFF figures, STROBE checklist).

All four points raised by Reviewer #1 have been addressed in the revised manuscript:

Confounding by indication: A dedicated paragraph in the Discussion now positions confounding by indication as the central interpretive caveat, acknowledging that BB suspension occurred exclusively in the context of cardiogenic or septic shock and that the observed associations likely reflect, in substantial part, the prognostic advantage of haemodynamic stability per se. E-value analyses have been added to quantify robustness to unmeasured confounding (conservative E-value ≥ 3.99 for the mortality finding).

Exposure definition and time-varying effects: The Limitations section now explicitly acknowledges that the binary exposure classification does not capture subsequent dose modifications or transient interruptions, introducing potential time-varying misclassification, and notes that a marginal structural model with time-updated IPTW would be the appropriate design in a larger sample.

Generalisability: The Discussion now explicitly distinguishes between transplant centres without access to durable ventricular assist devices — to which the present findings are most directly applicable — and higher-resource centres with routine VAD availability, where the clinical question changes materially and applicability remains to be established.

Clarity and readability: The Introduction and Discussion have been streamlined throughout, with redundant passages removed and the rationale refocused on the specific, under-studied scenario of prolonged concomitant BB and dobutamine therapy.

All major and minor points raised by Reviewer #2 have been addressed in the revised manuscript. A full point-by-point response is provided in the accompanying Response to Reviewers file. A summary of the key changes is as follows.

Major points:

Propensity model and confounding by indication: We clarify that the variables identified by the Reviewer — cardiogenic shock, IABP, dialysis, and dobutamine dose escalation — were confirmed by chart review to have occurred after the index date; their inclusion would therefore constitute mediator adjustment bias rather than confounder adjustment. This is now stated explicitly in the Methods, and Panel A/B of Table 1 separates baseline from post-index variables accordingly. LVEF and NT-proBNP were reserved for balance diagnostics and sensitivity analyses on grounds of parsimony (events-per-parameter ratio ≈ 2.3). Propensity score distributions by group are now displayed in Supplementary Figure S1. Confounding by indication is positioned as the central interpretive caveat throughout the Discussion.

Wide confidence intervals and sensitivity analyses: The primary sHR has been re-specified following unification of the weighting scheme (sHR HT 8.79, 95% CI 2.66–54.31; sHR death 0.077, 95% CI 0.014–0.192). Event counts are reported alongside every headline sHR. All four pre-specified sensitivity analyses are now reported in full in Supplementary Table S1 (Panel A). The Limitations explicitly acknowledge that the sample size precludes firm inference from the magnitude of the sHRs, and identifies the CIF-based absolute differences as the primary evidence.

Causal language and hypothesis-generating framing: All causal formulations have been removed. The Discussion interprets the early mortality divergence as reflecting two non-mutually exclusive phenomena (haemodynamic consequences of BB suspension and pre-existing shock severity), and acknowledges that the retrospective design cannot separate these effects. The Conclusion now reads: "these findings should be interpreted with caution and are best viewed as hypothesis-generating."

Minor points: All eleven minor points have been corrected: Abstract and Table 2 values are now concordant; weighting scheme descriptions are consistent throughout; exposure definition clarified (BB suspension documented ≥72 hours after the index date); Table 1 denominators corrected to n=18; TGO replaced with AST; Table 3 reference corrected to Table 2; covariate balance percentage corrected to 81.8% (9/11 variables); imbalanced variables correctly identified as LVEF and NT-proBNP; Figure 3 axis extended to display the full CI upper bound; British English applied consistently; STROBE checklist provided as S1 File.

The full itemised responses, with quoted passages from the revised manuscript, are in the Response to Reviewers document.

Attachment Submitted filename: Response_to_Reviewers_PONE-D-25-62240_2026-06-23 (3).docx

# Editor report: Decision Letter 1

**DOI:** 10.1371/journal.pone.0354128.r003

**Authors:** Moises Auron

5 Jul 2026

Continuation of Beta-Blockers During Prolonged Dobutamine Infusion in Heart Transplant–Prioritised Patients: A Competing-Risk Analysis

PONE-D-25-62240R1

Dear Dr. Rossi Neto,

We’re pleased to inform you that your manuscript has been judged scientifically suitable for publication and will be formally accepted for publication once it meets all outstanding technical requirements.

Within one week, you’ll receive an e-mail detailing the required amendments. When these have been addressed, you’ll receive a formal acceptance letter and your manuscript will be scheduled for publication.

An invoice will be generated when your article is formally accepted. Please note, if your institution has a publishing partnership with PLOS and your article meets the relevant criteria, all or part of your publication costs will be covered. Please make sure your user information is up-to-date by logging into Editorial Manager at Editorial Manager® and clicking the ‘Update My Information' link at the top of the page. For questions related to billing, please contact billing support.

If your institution or institutions have a press office, please notify them about your upcoming paper to help maximize its impact. If they’ll be preparing press materials, please inform our press team as soon as possible -- no later than 48 hours after receiving the formal acceptance. Your manuscript will remain under strict press embargo until 2 pm Eastern Time on the date of publication. For more information, please contact onepress@plos.org.

Kind regards,

Moises Auron, MD, FAAP, FACP, SFHM, FRCP (Lon), FRCPCH

Academic Editor

PLOS One

# Editor report: Acceptance letter

**DOI:** 10.1371/journal.pone.0354128.r004

**Authors:** Moises Auron

PONE-D-25-62240R1

PLOS One

Dear Dr. Rossi Neto,

I'm pleased to inform you that your manuscript has been deemed suitable for publication in PLOS One. Congratulations! Your manuscript is now being handed over to our production team.

At this stage, our production department will prepare your paper for publication. This includes ensuring the following:

* All references, tables, and figures are properly cited

* All relevant supporting information is included in the manuscript submission,

* There are no issues that prevent the paper from being properly typeset

You will receive further instructions from the production team, including instructions on how to review your proof when it is ready. Please keep in mind that we are working through a large volume of accepted articles, so please give us a few days to review your paper and let you know the next and final steps.

Lastly, if your institution or institutions have a press office, please let them know about your upcoming paper now to help maximize its impact. If they'll be preparing press materials, please inform our press team within the next 48 hours. Your manuscript will remain under strict press embargo until 2 pm Eastern Time on the date of publication. For more information, please contact onepress@plos.org.

You will receive an invoice from PLOS for your publication fee after your manuscript has reached the completed accept phase. If you receive an email requesting payment before acceptance or for any other service, this may be a phishing scheme. Learn how to identify phishing emails and protect your accounts at https://explore.plos.org/phishing.

If we can help with anything else, please email us at customercare@plos.org.

Thank you for submitting your work to PLOS ONE and supporting open access.

Kind regards,

PLOS ONE Editorial Office Staff

on behalf of

Dr. Moises Auron

Academic Editor

PLOS One
